# Residual Upper Arm Motor Function Primes Innervation of Paretic Forearm Muscles in Chronic Stroke after Brain-Machine Interface (BMI) Training

**DOI:** 10.1371/journal.pone.0140161

**Published:** 2015-10-23

**Authors:** Marco Rocha Curado, Eliana Garcia Cossio, Doris Broetz, Manuel Agostini, Woosang Cho, Fabricio Lima Brasil, Oezge Yilmaz, Giulia Liberati, Guilherme Lepski, Niels Birbaumer, Ander Ramos-Murguialday

**Affiliations:** 1 Institute of Medical Psychology and Behavioral Neurobiology, University of Tübingen, Silcherstr. 5, 72076, Tübingen, Germany; 2 International Max Planck Research School for Neural & Behavioral Sciences, Tübingen, Germany; 3 Edmond and Lily Safra International Institute of Neuroscience, Institute Santos Dumond, Natal, Brazil; 4 Institute of Neuroscience, Université catholique de Louvain, Louvain la Neuve, Belgium; 5 Department of Neurology, University of São Paulo, São Paulo, Brazil; 6 Department of Neurosurgery, University of Tübingen, Tübingen, Germany; 7 Ospedale San Camillo, IRCCS, Venice, Italy; 8 Institute for Diabetes Research and Metabolic Diseases, Helmholtz Center Munich, University of Tübingen, Tübingen, Germany; 9 TECNALIA Health Technologies, San Sebastian, Spain; Duke University, UNITED STATES

## Abstract

**Background:**

Abnormal upper arm-forearm muscle synergies after stroke are poorly understood. We investigated whether upper arm function primes paralyzed forearm muscles in chronic stroke patients after Brain-Machine Interface (BMI)-based rehabilitation. Shaping upper arm-forearm muscle synergies may support individualized motor rehabilitation strategies.

**Methods:**

Thirty-two chronic stroke patients with no active finger extensions were randomly assigned to experimental or sham groups and underwent daily BMI training followed by physiotherapy during four weeks. BMI sessions included desynchronization of ipsilesional brain activity and a robotic orthosis to move the paretic limb (experimental group, n = 16). In the sham group (n = 16) orthosis movements were random. Motor function was evaluated with electromyography (EMG) of forearm extensors, and upper arm and hand Fugl-Meyer assessment (FMA) scores. Patients performed distinct upper arm (e.g., shoulder flexion) and hand movements (finger extensions). Forearm EMG activity significantly higher during upper arm movements as compared to finger extensions was considered facilitation of forearm EMG activity. Intraclass correlation coefficient (ICC) was used to test inter-session reliability of facilitation of forearm EMG activity.

**Results:**

Facilitation of forearm EMG activity ICC ranges from 0.52 to 0.83, indicating fair to high reliability before intervention in both limbs. Facilitation of forearm muscles is higher in the paretic as compared to the healthy limb (p<0.001). Upper arm FMA scores predict facilitation of forearm muscles after intervention in both groups (significant correlations ranged from R = 0.752, p = 0.002 to R = 0.779, p = 0.001), but only in the experimental group upper arm FMA scores predict changes in facilitation of forearm muscles after intervention (R = 0.709, p = 0.002; R = 0.827, p<0.001).

**Conclusions:**

Residual upper arm motor function primes recruitment of paralyzed forearm muscles in chronic stroke patients and predicts changes in their recruitment after BMI training. This study suggests that changes in upper arm-forearm synergies contribute to stroke motor recovery, and provides candidacy guidelines for similar BMI-based clinical practice.

## Introduction

Stroke is one of the main causes of adult disability worldwide [1]. Motor impairment is one of the most common disabilities caused by stroke, decreasing quality of life as daily life activities are difficult to perform independently. Motor activity of the paretic limb modulates post-stroke plasticity and constitutes an important promoter of motor recovery [2]. Several studies have demonstrated that the practice of specific motor tasks can improve motor function in stroke patients even in the chronic phase, when spontaneous recovery is no longer expected [3–6]. However, as most studies have aimed to evaluate the effectiveness of distinct motor trainings to restore motor function, the biological mechanisms involved in motor recovery remain poorly explored. A better understanding of such mechanisms may help to improve both the prognosis of motor recovery and the development of individualized motor rehabilitation strategies.

Changes in motor function are associated with neuroplastic changes not only at the brain level but also at the level of the spinal cord. At the cortical level, motor recovery in stroke patients has been associated with functional reorganization in both contra- [6,7] and ipsilesional [2,6,7] hemispheres. Similarly, motor recovery after brain ischemia in animals is associated with increased neurite outgrowth and synaptogenesis in both contra- and ipsilesional hemispheres [8]. These findings provide evidence that cortical reorganization plays an important role in the restoration of motor function. At the level of the spinal cord, it was reported that after brain lesions reorganization of preserved corticospinal tract axons at the affected side of the spinal cord contribute to motor recovery in rodents [9,10]. These findings suggest that spinal cord reorganization also contributes to restoration of motor function.

Considering that neurons coding motor function are somatotopically organized in the cortex and spinal cord, and plastic mechanisms in both brain and spinal cord influence motor recovery after stroke, it is plausible that after stroke residual upper limb muscle activity indicates a functionally preserved neuronal population that can—through neuroplastic mechanisms—activate neurons coding motor function in the most paretic muscles and hence facilitate recruitment of these muscles. For instance, in patients with severe hand paresis residual upper arm motor function may indicate a functional neuronal population that can be recruited to activate neurons coding motor function in the paralyzed forearm muscles. Accordingly, it has been shown that among stroke patients with severe hand paresis, those presenting residual upper arm motor function are more likely to recover hand motor function as compared to those presenting severe upper arm paresis [11,12].

**Fig 1:**
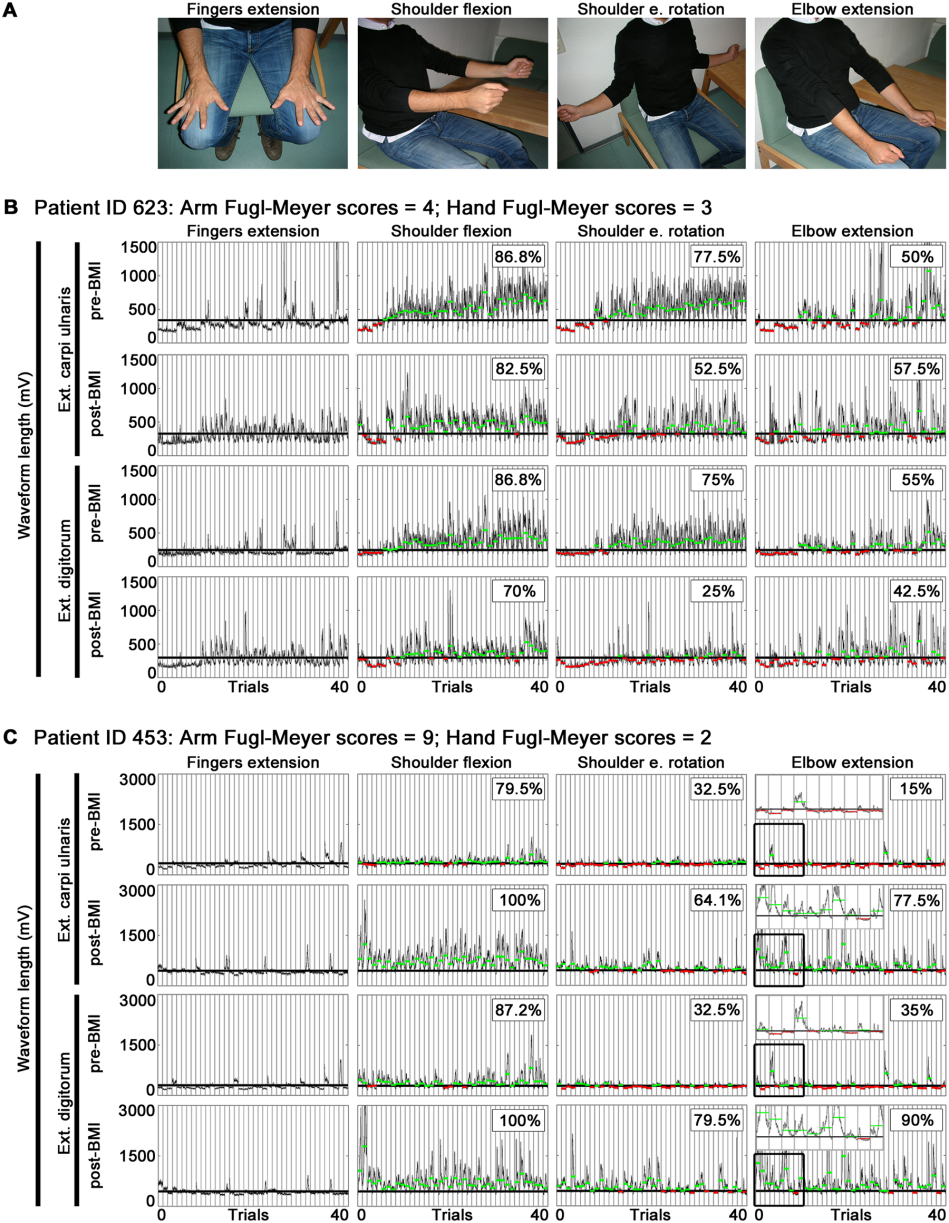
Forearm EMG activity in two stroke patients during finger extensions and upper arm movements. EMG activity from paretic forearm extensor carpi ulnaris and extensor digitorum during A) finger extensions and upper arm movements (shoulder flexion, shoulder external rotation and elbow extension) from two patients (ID 623 and ID 453) before and after Brain-Machine Interface (BMI) intervention. For each muscle and movement, waveform length was calculated within each trial (delimited by vertical lines). A threshold consisting of mean + 99% confidence interval of EMG waveform length from all trials during finger extensions is set for each patient in the paretic limb (thick black lines). This threshold is used during upper arm movements (thick black lines) to evaluate percentage of facilitation of forearm EMG activity across trials. Green lines represent mean– 99% confidence interval of EMG waveform length in each trial that is significantly higher than finger extensions threshold. Red lines represent mean– 99% confidence interval of EMG waveform length in each trial that is not significantly higher than finger extensions threshold. Percentage of facilitation of forearm EMG activity across trials is indicated at the top right in each upper arm movement. B) Patient ID 623 with poor upper arm FMA score (upper arm FMA score = 4, hand FMA score = 3); and C) Patient ID 453 with superior upper arm FMA score (upper arm FMA score = 9, hand FMA score = 2) before intervention. First ten trials during elbow extension in patient ID 453 are shown in detail (C). Ext. = Extensor. e. = external.

Neuroplastic reorganization of motor pathways after stroke are associated with the emergence of abnormal muscle synergies during performance of motor tasks [13–16]. For instance, chronic stroke patients with severe hand paresis were shown to activate completely paralyzed forearm muscles (e.g. forearm extensors) consistently stronger when performing upper arm movements (e.g., shoulder flexion) compared to hand movements alone (e.g. finger extensions)–an evidence of facilitation of forearm muscles activity during recruitment of upper arm muscles [17] (Fig 1). This finding is in line with a previous study indicating that upper arm movements are coupled with involuntary hand movements in stroke patients with moderate to severe paresis, a phenomenon that suggests a functional reconnection to forearm muscles dependent on recruitment of upper arm muscles and may have relevant implications in hand motor recovery [18].

To better understand the association between facilitation of forearm motor function and motor recovery after severe stroke it is relevant to evaluate the effects of rehabilitative interventions on facilitation of motor function. However, patients with severe hand paresis do not profit from current residual movement-based strategies as they are unable to perform the requested tasks. For those severely paretic patients we demonstrated in a randomized controlled trial that BMI-based motor rehabilitation linking intention to move the paretic limb to contingent visual and kinesthetic feedback significantly reduce motor impairment [6]. This finding is in line with previous single case studies [19–20] and is strenghtened by recent group studies [21,22] also reporting that BMI trainings improve motor function after severe stroke.

Considering the emergence of facilitation of forearm motor function in stroke patients with severe hand paresis [17] and the efficacy of BMI training to restore hand motor function in this patient population [6], we complemented these studies and hypothesized in the present *post hoc* study that residual upper arm function primes forearm muscle activity in severely paretic chronic stroke patients following BMI-based motor rehabilitation. We found that residual upper arm motor function positively influences the recruitment of forearm muscles after intervention and predicts changes (e.g. increases) in facilitation of forearm muscles after BMI-based intervention.

## Materials and Methods

### Patients

Thirty-two chronic stroke patients were included in the study. All patients fulfilled the following inclusion criteria: 1) no active finger extension in the paretic hand (modified Fugl-Meyer score for hand and finger mean±SE: 3,29±0,51; median: 2; range: 0–11) 2) time since stroke of at least 10 months 3) age between 18 and 80 years 4) no psychiatric or neurological condition other than stroke 5) no cerebellar lesion or bilateral motor deficit 6) ability to understand and follow instructions. Further inclusion criteria were described elsewhere [6]. All patients gave written consent before entry. The study was approved by the ethics committee of the Medical Faculty of the University of Tübingen.

### Experimental design and rehabilitative intervention

Rehabilitative intervention consisted of one hour of BMI training followed by one hour of behavioral physiotherapy every day (excluding weekends) over a four week period. BMI training involved the attachment of a hand or arm orthosis to the paretic upper limb, either to open and close the hand or extend and flex the arm respectively. All patients were instructed to try to move their paretic limb when using the orthotic-BMI system. Patients were randomly assigned to experimental or sham BMI groups: the experimental group received orthosis movement feedback contingent upon desynchronization of the ipsilesional EEG sensorimotor rhythm (SMR) (a cortical oscillatory activity associated with motor execution, planning or imagery [23]), whereas the sham group received random orthosis feedback, i.e. the movement of the orthosis was unrelated to brain activity. Only the experimental group showed significant improvements in upper arm and hand motor function after intervention, indicating that the engagement of brain activity in a motor task contingent to visual and kinesthetic feedback from paretic limb movement can promote motor recovery in chronic stroke patients with severe hand paresis (Further details of this study were published elsewhere [6]). Two patients from the sham group were discarded from analyses due to (1) equipament malfunctioning during BMI training (n = 1), and (2) faking severity of motor deficits to be included in the training (n = 1) [6]. Therefore, these patients were not included in the present study as well. As in our previous study [6], we analyzed patients in experimental (n = 16) and control (n = 14) groups. A demographics table is presented in Table 1.

**Table 1:** Demographics table with upper arm and hand Fugl-Meyer scores.

Group	ID	Age	Time since stroke (years)	Gender	Lesion location	hFMA Pre1/ Pre2/ Post	aFMA Pre1/ Pre2/ Post
experimental	48	45	4	M	subcortical	6 / 2 / 5	3 / 4 / 9
	89	51	3	F	subcortical	0 / 0 / 1	5 / 5 / 4
	154	62	4	M	subcortical	2 / 2 / 2	2 / 1 / 3
	155	30	5	F	subcortical	8 / 6 / 10	16/21/19
	258	47	10	M	subcortical	6 / 4 / 5	11/13/15
	261	68	4	F	subcortical	1 / 0 / 0	1 / 2 / 3
	263	34	1	F	mixed	4 / 3 / 6	4 / 8 / 10
	363	49	11	M	subcortical	2 / 5 / 2	6 / 4 / 7
	394	63	6	M	subcortical	0 / 1 / 2	6 / 4 / 7
	400	39	12	M	mixed	8 / 7 / 9	17/16/17
	453	32	2	F	mixed	2 / 2 / 2	9 / 9 / 13
	523	39	13	F	mixed	2 / 3 / 3	2 / 4 / 4
	554	52	4	F	subcortical	6 / 9 / 8	6/12/16
	563	40	7	M	subcortical	2 / 3 / 3	7/12/13
	615	26	2	M	mixed	2 / 1 / 3	11/16/15
	623	68	2	M	mixed	2 / 3 / 4	7 / 4 / 7
sham	35	61	7	M	mixed	2 / 2 / 3	23/25/19
	207	36	4	M	subcortical	11 / 8 / 8	21/21/21
	241	36	4	M	mixed	3 / 2 / 4	1 / 1 / 1
	510	44	10	M	mixed	3 / 8 / 11	11/10/15
	516	51	2	F	subcortical	3 / 4 / 3	13/15/9
	533	32	18	M	subcortical	11/11/11	24/21/23
	536	50	1	M	mixed	0 / 0 / 1	24/21/23
	551	62	4	M	mixed	2 / 4 / 2	5 / 2 / 4
	578	28	19	M	mixed	4 / 2 / 2	10/11/8
	593	71	2	F	mixed	1 / 0 / 0	1 / 0 / 0
	610	56	2	M	mixed	3 / 2 / 1	6 / 6 / 3
	612	53	1	F	mixed	2 / 1 / 1	7 / 6 / 12
	613	64	2	F	mixed	3 / 1 / 4	14/15/17
	622	54	1	F	subcortical	0 / 0 / 1	1 / 0 / 1

FMA = Fugl-Meyer assesment. hFMA = hand FMA scores. aFMA = upper arm FMA scores

Motor function assessments were performed twice before intervention (two months before and one day before the first session, referred from now on as “Pre1” and “Pre2” respectively) and once immediately after intervention (“Post”). For comparison with post-intervention assessment scores, both Pre1 and Pre2 measurements were averaged and used as baseline to reduce variability.

### Assessment of motor function

#### 1) Fugl-Meyer assessment

A modified version of Fugl-Meyer assessment (FMA) for the upper limb was used to evaluate motor function. The only modifications from the original upper limb FMA scores are the exclusion of scores related to (1) coordination and speed, and (2) reflexes. These changes were due to: (1a) analysis of upper arm and hand subscores separately, what according to Fugl-Meyer’s report [12] do not include coordination/speed scores, (1b) inability of the patients to perform necessary movements to measure coordination and speed (e.g. touch their noses with the index finger fully extended), because patients in the study had no remaining finger extension (inclusion criteria), (2a) “normal reflex activity” scores assessment is recommended to be performed only if patients achieve 6 points in Fugl-Meyer scale stage IV [12], and (2b) decreasing uncertainty in our analyses, as assessment of reflexes is reported to introduce unreliability in the analyses [24, 25]. In FMA, a higher score indicates better motor performance [12]. Two professional physiotherapists assessed all patients, and each patient was assessed by the same physiotherapist during all sessions.

#### 2) Facilitation of forearm motor function: Electromyographic (EMG) recordings

Patients were seated comfortably with both arms positioned over the legs and eight bipolar electromyogram (EMG) electrodes were placed in each upper limb on top of the following muscles: 1) extensor carpi ulnaris 2) extensor digitorum 3) flexor carpi radialis, palmaris longus, flexor carpi ulnaris 4) biceps 5) triceps 6) anterior deltoid 7) lateral deltoid 8) posterior deltoid and infraspinatus [6]. Patients were instructed to perform isometric muscular contractions (i.e. maintain posture) with both upper limbs at the end of six movements: 1) shoulder flexion; 2) shoulder external rotation; 3) elbow extension; 4) supination of the arm; 5) wrist extension; and 6) finger extensions. These movements were selected based on their similarity to movements performed during FMA for upper limb motor scoring (more information can be found elsewhere [6]). Patients were instructed to avoid compensatory movements. To control for changes in EMG position (and changes in EMG activity due to electrode position) between sessions, a surgical skin marker was used routinely to re-draw electrodes contour. To avoid changes in EMG activity due to changes in temperature, all EMG measurements were performed in the same room with controlled temperature.

Each measurement consisted of three or four blocks composed of sixty trials (ten trials per movement in each block). Each movement was performed with both upper limbs after a visual and auditory instruction period of 6 secs. Immediately after the instruction period a “GO” cue was presented and an action period of 6 secs started. During this period patients were instructed to perform one of the six movements to reach a target position, where they had to maintain the posture to achieve isometric contraction of muscles. A second cue “END” indicated the end of the trial and the start of an inter-trial interval lasting between 4 and 7 sec.

The EMG signal was acquired at 2500 Hz, high-pass filtered at 10Hz and notch filtered at 50Hz. Data were bipolarized, rectified and artifacts were rejected after visual inspection. Data were epoched from -9 to 9 sec relative to the “GO” cue. To avoid changes in EMG activity due to inter- and intra-session variability, EMG signals in each trial were baseline corrected with the averaged signal from -7 to -2 sec before the “Go” cue, i.e. mean baseline EMG activity during each rest window (between -7 and -2 sec) was subtracted from EMG activity in the following activity window [17]. Independent component analysis was applied in each task and the eight principal components were visually inspected for artifact rejection. A 200 msec sliding window with 20 msec overlap was used to calculate the EMG waveform length, a measurement of EMG amplitude and frequency [26]. Waveform length indicates the complexity of the EMG waveform and is calculated by:]

$$WL=\sum_{k=1}^{L}\left|x_{k}-x_{k-1}\right|$$

where “*L*” is the window length applied for the waveform length (*WL*) estimation and “*x*” is the amplitude of the EMG signal at time point “*k*”.

We defined “facilitation of forearm motor function” (or “facilitation of forearm EMG activity”) as the influence exerted by the recruitment of proximal muscles during upper arm movements on forearm muscle activity. To measure facilitation of forearm EMG activity, forearm EMG waveform length values during finger extensions were used to compute a distribution of EMG activity during contraction of forearm extensor muscles, and this was used to calculate forearm extensors EMG activity thresholds. Forearm EMG thresholds were used as reference for forearm EMG activity during finger extensions, when there is no engagement of upper arm motor function. In each patient two forearm muscles (extensor carpi ulnaris and extensor digitorum) were used to determine two thresholds in each arm, one per muscle. Each threshold consisted of mean + 99% of confidence interval (CI) from EMG waveform length from all trials of the respective muscle during finger extensions.

Forearm EMG thresholds were used to compare forearm EMG activity during finger extensions (i.e., movements without the recruitment of upper arm muscles) and during upper arm movements (movements with the recruitment of upper arm muscles, i.e., shoulder flexion, shoulder external rotation, and elbow extension). Forearm EMG thresholds were compared against mean—99% CI from EMG waveform length from the same forearm muscle in each trial during upper arm movements (shoulder flexion, shoulder external rotation and elbow extension). The percentage of trials, in which the mean—99% CI from forearm EMG waveform length during a particular upper arm movement was higher than the respective threshold, was calculated for each forearm muscle separately (extensor carpi ulnaris and extensor digitorum). Facilitation of forearm EMG activity was calculated as:

$$ffEMG=\frac{100}{N}\sum_{i=1}^{N}\theta \left(x_{i}-y\right)$$

where “*ffEMG*” is facilitation of forearm EMG activity, “*N*” is the number of trials, and “*θ(x*_*i*_ *−y)*” is the Heaviside function defined as:

$$\theta \left(x_{i}-y\right)=\{\begin{array}{c} 1,x\geq y\\ 0,x<y \end{array}$$

where “x” is the mean + 99% CI EMG activity during trial “*i*”, and “*y*” is the muscle threshold, i.e. mean– 99% CI EMG activity of all trials during finger extensions.

This measure indicated the amount of facilitation of EMG activity in each forearm muscle during recruitment of upper arm muscles. Facilitation of forearm EMG activity was analyzed separately for each upper arm movement. Fig 1 shows examples of forearm EMG activity during finger extensions and upper arm movements in two patients with severe hand paresis before intervention: one presenting residual upper arm function (Fig 1B), and one presenting severe upper arm paresis (Fig 1C).

We excluded movement 4 (arm supination) from analyses as both forearm and upper arm muscles are engaged during this movement; and we excluded movement 5 (wrist extension) to avoid uncertainty in the analysis, as we did not control for residual wrist extension capacity before study admission.

### Statistical analysis

Factorial ANOVA with repeated measures was performed to compare thresholds or facilitation of forearm EMG activity between sessions (Pre1, Pre2 and Post) and groups (experimental and control). Intraclass correlation coefficient (ICC) was performed in sessions Pre1 and Pre2 to evaluate reliability of the percentage of facilitation of forearm EMG activity before intervention [27]. Specifically, we calculated the ICC model that determines the degree of absolute agreement for averages based on independent measurements [27].…. It is considered that ICC > 0.75 represents excellent reliability, 0.4 < ICC < 0.75 represents moderate to good reliability, and 0.4 < ICC represents poor reliability [28]. Spearman’s rank correlation was used to evaluate the relationship between the ordinal upper arm FMA or hand FMA scores and percentage of facilitation of forearm EMG activity. Bonferroni correction was used to control for multiple (eighteen) comparisons, with the adjusted *p* <0.0028 being considered significant. Unpaired *t* tests were performed with percentage of facilitation of forearm EMG activity between the healthy and paretic limbs. Values are given as means ± standard errors.

## Results

### Reliability of facilitation of forearm EMG activity

To evaluate the reliability of the percentage of facilitation of forearm EMG activity across trials before intervention, we analyzed ICC on Pre1 and Pre2 sessions with all patients grouped together (n = 30). Percentage of facilitation of forearm EMG activity showed moderate to excellent reliability in both paretic (0.52 ≤ ICC ≤ 0.76; S1 Table) and healthy limbs (0.53 ≤ ICC ≤ 0.83; S1 Table).

### Forearm EMG thresholds

To evaluate whether there were significant changes on muscle thresholds between sessions (Pre1, Pre2 and Post) and groups (experimental and sham), a two-way ANOVA with repeated measures was performed separately for thresholds from each muscle (extensor ulnaris and extensor digitorum). We found no significant main effect or sessions x groups interaction for either the extensor carpi ulnaris (main effect “sessions”: F_(2,26)_ = 2.774, *p* = 0.76; main effect “groups”: F_(1,13)_ = 0.124, *p* = 0.73; interaction: F_(2,26)_ = 1.079; *p = *0.35) or the extensor digitorum (main effect “sessions”: F_(2,26)_ = 1.461, *p* = 0.25; main effect “groups”: F_(1,13)_ = 0.087, *p* = 0.77; interaction: F_(2,26)_ = 1.304; *p* = 0.88).

### Facilitation of forearm EMG activity

Percentages of facilitation of forearm EMG activity for both paretic and healthy limbs before and after intervention are shown in Table 2. Before intervention, mean percentage of facilitation of EMG activity in paretic forearm muscles during upper arm movements trials ranged from 43.23±7.34% to 66.61±8.49% in the experimental group and from 42.72±9.7% to 69.11±8.8% in the sham group. After intervention, mean percentage of facilitation of EMG activity in paretic forearm muscles during upper arm movements trials ranged from 47.77±8.67% to 73.82±7.71% in the experimental group and from 52.53±11.06% to 68.57±9.57% in the sham group. In the healthy limb, mean percentage of facilitation of forearm EMG activity during upper arm movements ranged from 1.41±0.85% to 21.02±7.91% in the experimental group and from 3.51±1.87% to 26.96±11.02% in the sham group before intervention, while after intervention ranged from 1.76±0.76% to 13.41±7.38% in the experimental and from 3.99±1.86% to 19.85±9.14% in the sham group (Table 2).

**Table 2:** Facilitation of forearm EMG activity before (Pre1 and Pre2) and after (Post1) intervention in experimental and sham groups.

		Percentage of facilitation of forearm EMG activity (%)					
Forearm	Upper arm	Experimental group			Sham group		
muscle	movement	Pre1	Pre2	Post1	Pre1	Pre2	Post1
paretic							
Ext. ulnaris	Sh. flexion	66.61±8.49	60.94±8.73	73.82±7.71	56.5±9.7	69.11±8.8	65.72±10.19
	Sh. rotation	56.98±6.82	62.36±7.77	59.9±7.77	51.1±8.98	59.99±8.07	56.87±10.39
	Elb. extension	56.32±9.16	58.62±7.2	69.11±8.34	47.76±6.67	63.83±6.9	52.53±11.06
Ext. digitorum	Sh. flexion	57.71±8.06	60.43±9.76	61.72±9.46	52.48±9.3	60.38±9.59	68.57±9.57
	Sh. rotation	43.23±7.34	55.2±9.43	47.77±8.67	42.72±9.7	44.84±8.76	55.23±10.82
	Elb. extension	57.09±8.4	56.23±6.6	67.91±8.43	48.05±8.34	59.49±8.45	55.4±10.89
healthy							
Ext. ulnaris	Sh. flexion	15.79±8.19	9.61±3.55	8.51±3.57	10.36±6.57	20.6±9.22	17.99±9.23
	Sh. rotation	20.68±8.22	21.02±7.91	13.41±7.38	12.09±6.64	26.96±11.02	14.6±6.46
	Elb. extension	8.93±4.23	7.08±5.14	1.76±0.76	5.89±3.66	5.58±2.75	5.3±2.74
Ext. digitorum	Sh. flexion	7.99±3.68	7.37±4.03	6.99±3.52	15.54±8.29	19.54±8.91	19.85±9.14
	Sh. rotation	2.66±2.09	1.41±0.85	8.12±5.42	12.54±8.62	17.28±8.77	9.41±6.85
	Elb. extension	3.75±2.03	9.54±4.72	5±3.77	3.51±1.87	5.38±2.81	3.99±1.86

Values are given as mean±standard error. Ext. = extensor, Sh. = Shoulder, Elb. = Elbow.

To evaluate whether there were significant changes on percentage of facilitation of forearm EMG activity between arms (paretic and healthy), upper limb movements (shoulder flexion, shoulder rotation and elbow extension) and muscles (extensor carpi ulnaris and extensor digitorum) across sessions (Pre1, Pre2 and Post), a four-way ANOVA with repeated measures was performed separately for each group (experimental and sham). We found no significant interactions between independent variables in any group. In both groups, only main effect of “arm” was significant (experimental: F_(1,15)_ = 102.677, p<0.001; sham: F_(1,13)_ = 75.89, p<0.001).

### Facilitation of forearm EMG activity between paretic and healthy limbs

To analyze whether facilitation of forearm EMG activity reflects adaptive plasticity associated with limb paresis after stroke, we compared the percentage of facilitation of forearm EMG activity between paretic and healthy arms in both experimental and sham groups before intervention. Percentage of facilitation of forearm EMG activity was significantly higher in the paretic limb as compared to the healthy limb in both experimental and control groups during all upper arm movements (Table 3).

**Table 3:** Unpaired t tests between facilitation of forearm EMG activity (%) in the paretic and healthy arms.

		Experimental group			Sham group		
		Facilitation of EMG activity (%)			Facilitation of EMG activity (%)		
Forearm	Upper arm	paretic	healthy		paretic	healthy	
threshold	movements	limb	limb	p value	limb	limb	p value
Ext. ulnaris	Sh. flexion	63.78±6.71	12.7±4.52	<0.001	62.8±8.15	15.48±7.33	<0.001
	Sh. rotation	59.67±5.79	20.85±7.41	<0.001	55.55±7.62	19.53±8.39	0.004
	Elb. extension	57.47±7.75	8±4.47	<0.001	55.79±5.76	5.74±2.37	<0.001
Ext. digitorum	Sh. flexion	59.91±7.48	7.68±3.51	<0.001	56.43±8.23	17.54±7.95	0.002
	Sh. rotation	49.21±6.71	2.03±1.16	<0.001	43.78±7.88	14.91±7.82	0.015
	Elb. extension	56.66±6.78	6.64±3.11	<0.001	53.77±7.43	4.45±1.84	<0.001

Ext. = extensor, Sh. = shoulder, Elb. = elbow.

### Correlation between facilitation of forearm EMG activity and upper arm or hand FMA scores

To investigate the influence of upper arm or hand motor impairment on facilitation of forearm EMG activity, we correlated upper arm FMA and hand FMA scores before intervention with percentage of facilitation of forearm EMG activity across trials before, after and with the difference between before and after intervention in both experimental and sham groups. All correlations are summarized in Table 4. Individual values of percentage of facilitation of forearm EMG activity pre- and post-intervention are shown in S2 Table.

**Table 4:** Spearman correlations between upper arm or hand FMA scores and facilitation of forearm EMG activity.

			Pre training							
			Experimental group				Sham group			
	Forearm	Upper arm	Hand FMA		Arm FMA		Hand FMA		Arm FMA	
Session	muscle	movement	R	p	R	p	R	p	R	p
Pre	Ext. ulnaris	Sh. flexion	0.267	0.32	0.29	0.28	0.464	0.09	0.478	0.08
		Sh. rotation	0.019	0.95	0.112	0.68	0.285	0.32	0.551	0.04
		Elb. extension	**0.697**	**0.0027**	0.448	0.08	0.523	0.06	0.732	0.003
	Ext. digitorum	Sh. flexion	0.072	0.79	0.305	0.25	0.536	0.05	0.644	0.01
		Sh. rotation	-0.21	0.44	0.106	0.7	0.364	0.2	0.553	0.04
		Elb. extension	0.568	0.02	0.441	0.09	0.704	0.005	**0.793**	**<0.001**
Post	Ext. ulnaris	Sh. flexion	0.458	0.07	0.552	0.03	0.515	0.06	0.637	0.01
		Sh. rotation	0.321	0.23	**0.753**	**<0.001**	0.533	0.05	**0.749**	**0.002**
		Elb. extension	0.684	0.003	0.55	0.03	0.463	0.1	0.595	0.02
	Ext. digitorum	Sh. flexion	0.331	0.21	0.441	0.09	0.440	0.12	**0.779**	**0.001**
		Sh. rotation	0.251	0.35	0.643	0.007	0.492	0.07	**0.752**	**0.002**
		Elb. extension	0.686	0.003	**0.732**	**0.001**	0.408	0.15	0.7	0.005
Delta	Ext. ulnaris	Sh. flexion	0.245	0.36	0.302	0.26	0.311	0.28	0.623	0.02
		Sh. rotation	0.327	0.22	**0.709**	**0.002**	0.32	0.26	0.52	0.06
		Elb. extension	0.074	0.78	0.178	0.51	0.219	0.45	0.33	0.25
	Ext. digitorum	Sh. flexion	0.507	0.04	0.456	0.08	-0.13	0.65	0.198	0.5
		Sh. rotation	0.382	0.14	**0.827**	**<0.001**	0.088	0.76	0.529	0.05
		Elb. extension	0.311	0.24	0.497	0.05	0.106	0.72	0.498	0.07

Significant values are adjusted for 18 comparisons (p<0.0028) and are presented in bold. Ext. = extensor, Sh. = shoulder, Elb. = elbow. Delta = Post-Pre sessions.

**Fig 2:**
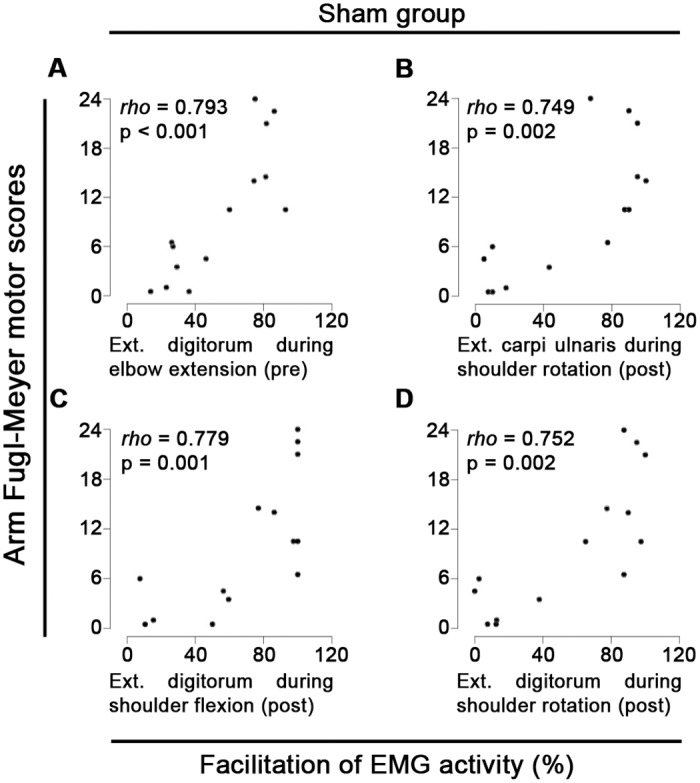
Upper arm Fugl-Meyer scores predict facilitation of forearm EMG activity—sham group. Spearman correlations between upper arm Fugl-Meyer assessment (FMA) motor scores before intervention and percentage of facilitation of electromyographic (EMG) activity from (A) extensor carpi ulnaris during elbow extension before intervention; (B) extensor carpi ulnaris during shoulder external rotation, (C) extensor digitorum during shoulder flexion, and (D) extensor digitorum during shoulder external rotation after intervention. Ext = Extensor.

Before intervention the experimental group presented significant positive correlation between percentage of facilitation of EMG activity in the extensor carpi ulnaris muscle and hand FMA scores (R = 0.627, *p* = 0.0027), while the sham group presented significant positive correlation between percentage of facilitation of EMG activity in the extensor digitorum muscle and upper arm FMA scores (R = 0.793, *p*<0.001, Fig 2A), both during elbow extension.

**Fig 3:**
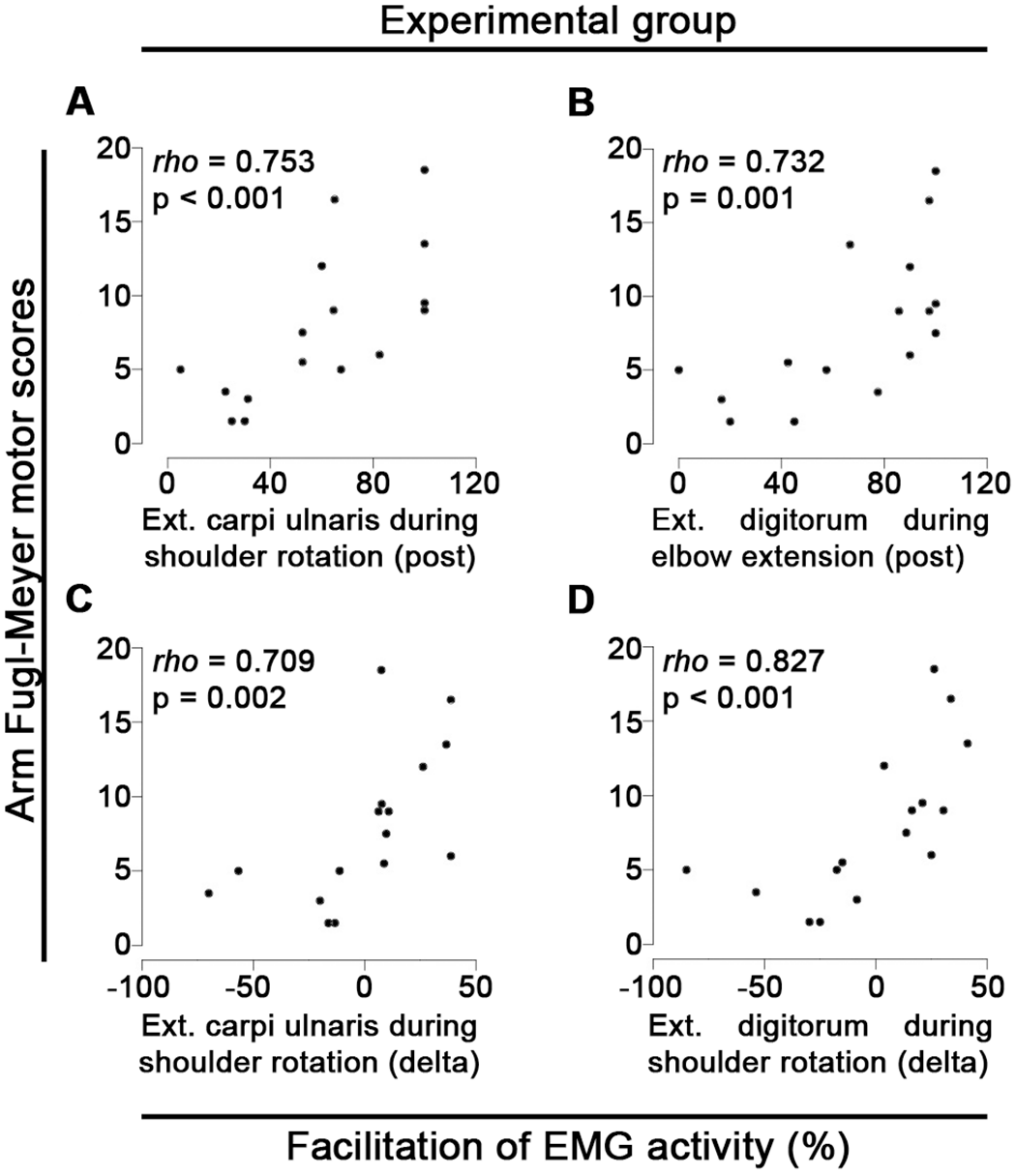
Upper arm Fugl-Meyer scores predict changes in facilitation of forearm EMG activity—experimental group. A, B) Spearman correlations between upper arm Fugl-Meyer assessment (FMA) motor scores before intervention and percentage of facilitation of forearm electromyographic (EMG) activity after intervention from: (A) extensor carpi ulnaris during shoulder external rotation, (B) extensor digitorum during elbow extension. C, D) Spearman correlations between upper arm FMA motor scores and changes in percentage of facilitation of forearm EMG activity (delta, i.e. post—pre sessions) from: (C) extensor carpi ulnaris, (D) extensor digitorum during shoulder external rotation. Ext = Extensor.

Upper arm FMA scores are significantly positively correlated with percentage of facilitation of EMG activity after intervention on extensor carpi ulnaris muscle during shoulder external rotation (R = 0.753, *p*<0.001, Fig 3A) and on extensor digitorum muscle during elbow extension (R = 0.732, *p* = 0.001, Fig 3B) in the experimental group. In the sham group upper arm FMA scores are significantly positively correlated with percentage of facilitation of EMG activity after intervention on extensor carpi ulnaris muscle during shoulder external rotation (R = 0.749, *p* = 0.002, Fig 2B), and on extensor digitorum muscle during shoulder flexion (R = 0.779, *p* = 0.001, Fig 2C) and shoulder external rotation (R = 0.752, *p* = 0.002, Fig 2D). Noteworthy, several patients showed stronger forearm EMG activity during upper arm movements as compared to finger extensions in 100% of trials after intervention (S2 Table). To illustrate this point, when compared to finger extensions, the extensor carpi ulnaris activity was stronger in 100% of trials for 5/16 patients from the experimental and 4/14 patients from the sham group during shoulder flexion, 4/16 patients from the experimental and 1/14 patients from the sham group during shoulder external rotation, and 3/16 patients from the experimental and 1/14 patients from the sham group during elbow extension after intervention. Similarly, compared to finger extensions, the extensor digitorum after intervention was stronger in 100% of trials for 3/16 patients from the experimental and 5/14 patients from the sham group during shoulder flexion, 2/16 patients from the experimental and 1/14 patients from the sham group during shoulder external rotation, and 3/16 patients from the experimental and 2/14 patients from the sham group during elbow extension (S2 Table).

However, in the experimental group only upper arm FMA scores were significantly correlated with changes in percentage of facilitation of forearm EMG activity (post—pre intervention values). Specifically, upper arm FMA scores correlated with both forearm extensor muscles during shoulder external rotation (extensor carpi ulnaris: R = 0.709, p = 0.002, Fig 3C; extensor digitorum: R = 0.827, p<0.001, Fig 3D). In the sham group no significant correlation was found between upper arm or hand FMA scores and changes in percentage of facilitation of forearm EMG activity.

## Discussion

In the present study we demonstrated in stroke patients with severe hand paresis (no active finger extension) that upper arm FMA scores: 1) predict facilitation of forearm EMG activity after Brain-Machine Interface (BMI)-based intervention, 2) is a superior predictor of facilitation of forearm EMG activity compared to hand FMA scores, and 3) predict changes (e.g., increases) in the recruitment of forearm muscles during upper arm movements after BMI-based intervention. Altogether, our findings indicate that after severe stroke residual upper arm motor function positively influences the recruitment of paralyzed forearm muscles and is relevant for increasing recruitment of these muscles during upper arm movements after a BMI-based intervention. A better understanding of the coupling between residual upper arm motor function and forearm motor activity in stroke patients with severe hand paresis may contribute to tailoring motor rehabilitation interventions to individual patients and to predicting outcomes after intervention. Moreover, it contributes to guide the selection of patients in future studies and provide candidacy guidelines for similar BMI-based clinical practice. This is specially relevant in stroke patients with severe hand paresis (as in the current study) because they are unable to actively extend the fingers and hence are unable to perform functional motor tasks required in conventional physiotherapies and constraint-induced therapies.

We demonstrated in a test-retest evaluation that facilitation of forearm EMG activity—measured as the percentage of trials during which EMG waveform length in forearm muscles is significantly higher during upper arm movements as compared to finger extensions—is a reliable event in both paretic and healthy arms of stroke patients. Moreover, we found that percentage of facilitation of forearm motor function is significantly higher in the paretic limb as compared to the healthy limb. This finding suggests that an increased facilitation of forearm muscles in the paretic limb reflects a reliable abnormal muscle synergy after stroke [13–16,18].

We found in both experimental and sham groups that upper arm FMA scores predict facilitation of forearm EMG activity in a single muscle and during a single upper arm movement before intervention, what indicates a low predictive capacity for facilitation of forearm EMG activity before intervention. However, upper arm FMA scores pre-intervention significantly predict facilitation of EMG activity in both forearm muscles (extensor carpi ulnaris and extensor digitorum) and during distinct upper arm movements in both experimental and sham groups after intervention, indicating an increase in predictive capacity for facilitation of forearm EMG activity after intervention. These findings indicate an increase in the association between residual upper arm motor function before intervention and recruitment of forearm muscles after motor training. Comparatively, hand FMA scores before intervention did not show major changes in predictive capacity of facilitation of forearm EMG activity after intervention. Accordingly, patients presenting superior upper arm FMA scores from both sham (e.g., ID35, ID207 and ID533) and experimental (e.g., ID155, ID554 and ID615) groups presented 100% of facilitation of forearm EMG activity after intervention (Figs 2 and 3). Interestingly, this pattern seems to be independent of hand FMA scores before intervention. For instance, patients with superior upper arm FMA scores presented 100% of facilitation of forearm EMG activity after intervention independent of poor (e.g., ID615 and ID35) or superior (e.g., ID554 and ID533) hand FMA scores. Altogether, these findings indicate that upper arm FMA scores are a reliable indicator of functional connection to forearm paretic muscles, and suggest that upper arm FMA scores are a superior predictor of the patient’s capacity to recruit forearm muscles during upper arm movements as compared to hand FMA scores.

Furthermore, in the experimental group only upper arm FMA scores pre-intervention positively predict changes (i.e. post—pre intervention) in facilitation of EMG activity in both forearm muscles, specifically during shoulder external rotation. This finding implies that the better the residual upper arm motor function before intervention, higher is the increase in facilitation of forearm muscles activity after BMI-based motor rehabilition, and suggests a functional reconnection to forearm muscles dependent upon neurons associated with upper arm function. Hence, it provides a physiological basis to explain why stroke patients exhibiting severe hand paresis and residual upper arm function have a better prognosis for hand motor recovery as compared to patients with severe hand and upper arm paresis [11,12]. Accordingly, individual patients from sham group presenting superior upper arm FMA scores before intervention may be benefiting from random feedback as well as behavioral physiotherapy to achieve 100% of facilitation of forearm EMG activity after intervention (e.g., ID35, ID207 and ID533).

The current findings complement our previous findings indicating that only the experimental group significantly improved hand FMA scores after BMI training [6] and strengthens the relevance of neuroprosthetic training with contingent feedback to improve hand motor function in severely paretic chronic stroke patients. Since only in the experimental group (1) upper arm FMA scores before intervention significantly predict changes in facilitation of forearm EMG activity after intervention, and (2) there was a significant increase in hand FMA scores after intervention [6], it is plausible that rehabilitation strategies involving simultaneous engagement of paretic arm and hand in multi-joint movements facilitates forearm and hand motor recovery in stroke patients with severe hand paresis. However, further studies are needed to explore the contribution of facilitation of forearm muscles to motor recovery after stroke.

Previous studies demonstrated that changes in surface EMG features (e.g. muscle contractions [6, 29–30], agonist-antagonist muscles co-contraction [29] or other complex muscular processes [31]) are associated with clinical changes in motor function of stroke patients. However, a frequent caveat regarding analysis of longitudinal EMG assessments is that changes in EMG electrodes placement and impedance across sessions influence recorded EMG activity. In our study, since we redrew contours every day around electrode pads and EMG impedance was controlled to similar values across sessions, we believe that electrode positions and impedance were fairly controlled and do not account for the reported changes in EMG activity.

Abnormal synergies may worsen motor performance due to uncontrolled co-contraction of muscles related to the task, but will not interfere with motor performance when recruiting muscles unrelated to the performance [32]. We estimate that an increased recruitment of forearm extensors while performing upper arm movements (e.g., shoulder flexion, external rotation or elbow extension) does not interfere with the performance of these movements, as forearm extensors are not agonist or antagonist muscles associated with them. Thus, it implies that facilitation of forearm motor function is not maladaptive plasticity worsening motor function. Instead, it may represent a relevant phenomenon to be explored for severe stroke motor recovery [14, 18, 33], e.g. by strenghtening forearm extensors activity during upper arm movements in Myoelectric-Computer Interface trainings [34].

Noteworthy, although we have here described increase in facilitation of forearm EMG activity after BMI-based motor rehabilitation, this phenomenon may be occurring in severely paretic chronic stroke patients after forearm and hand motor recovery in general, and we have no evidence linking this phenomenon specifically to BMI trainings. However, as these patients do not profit from conventional physiotherapies due to severity of the paresis, BMI trainings play an important role on investigations of the association between facilitation of forearm EMG activity and motor recovery in severely paretic stroke patients as BMI-based interventions are being demonstrated to restore motor function in this patient population [6, 21–22]. Moreover, for studies focusing on stroke motor recovery based on other strategies (e.g., constraint-induced therapies), it is an open question whether facilitation of forearm EMG activity is detectable as patients eligible to undergo those strategies have mild to moderate paresis and hence superior voluntary forearm muscle activity as compared to the patients analyzed in the present study.

## Conclusions

We conclude that residual upper arm function positively influences the recruitment of forearm muscles during upper arm movements in severe chronic stroke patients, and this influence may contribute to forearm motor recovery after BMI training. Therefore our results provide candidacy guideline for similar BMI-based studies and clinical practice, and support neuroprosthetic-based rehabilitation strategies engaging simultaneously upper arm and forearm muscles, in order to facilitate forearm muscles recruitment and recovery in chronic stroke patients with severe hand paresis.

## Supporting Information

S1 Table Intraclass correlation coefficient of percentage of facilitation of forearm EMG activity pre- and post-intervention. (XLSX)

S2 Table Facilitation of EMG activity in the paretic limb of patients from experimental and sham groups. (XLSX)
